# A nonvolatile bidirectional reconfigurable FET based on S/D self programmable floating gates

**DOI:** 10.1371/journal.pone.0284616

**Published:** 2023-05-24

**Authors:** Xiaoshi Jin, Shouqiang Zhang, Xi Liu

**Affiliations:** School of Information Science and Engineering, Shenyang University of Technology, Shenyang, China; University of Kashmir, INDIA

## Abstract

A nanoscale nonvolatile bidirectional reconfigurable field effect transistor (NBRFET) based on source /drain (S/D) self programmable floating gates is proposed. Comparing to the conventional reconfigurable field effect transistor (RFET) which requires two independently powered gates, the proposed NBRFET requires only one control gate. Beside, S/D floating gates are introduced. Reconfigurable function is realized by programming different types of charges into the S/D floating gates through biasing the gate at a positive or negative high voltage. The effective voltages of the S/D floating gates are determined jointly by the quantity of the charge stored in the S/D floating gates and the gate voltage. In addition, the charge stored in the floating gate has an effect of reducing the energy band bending near the source/drain regions when the gate is reversely biased, thereafter, the band to band tunneling (BTBT) leakage current can be largely decreased. The scale of the proposed NBRFET can be reduced to nanometer level. The device performances such as the transfer and output characteristics are verified by device simulation, which proves that the proposed NBRFET has very good performance in the nanometer scale.

## Introduction

The smallest unit of CMOS integrated circuit is MOSFET [1,2]. With the continuous reduction of MOSFET size and the increasingly physical limit of photolithography technology, in order to continue Moore’s law, new technologies must be tried to develop [3]. Although in recent years, research on quantum computing and integrated circuit devices based on new materials and the development of related integrated circuits has been ongoing [4,5], however, as the mainstream silicon-based technology, due to its mature process technology, various new ideas for improvement also emerge in endlessly. Reducing the number of devices required for logic function modules is a soft way to improve the integration of integrated circuits under the restriction that lithography technology cannot be further broken through. The reconfigurable field effect transistor (RFET) aroused the attention of the academic community in recent years. It can actually be seen as an expanded application of dopingless and electrostatic doped based devices [6–9]. However, compared with today’s mainstream technology, the reported RFET size is much larger. However, compared with the current technology, the reported RFET size is much larger. At nanoscale size, it is still unclear whether RFET can work properly like the FinFET technology [10–13]. RFET usually does not need to be doped, and its source and drain electrons or holes are generated by applying positive or negative voltage to the programming gate and through tunneling effect [14–16]. Therefore, comparing to the mainstream technology, RFET needs to add a program gate. The program gate makes the interconnection more complicated. For the device whose size has been reduced to nanometer level, if RFET is reversely biased, the energy band bending will be enhanced due to that the program gate is continuously positively or negatively biased, resulting in the band to band tunneling and leakage current is increased. To reduce the leakage current, we have proposed a nanoscale nonvolatile bidirectional RFET (NBRFET) based on S/D self programmable floating gates in this work. Comparing to conventional RFET which requires two independently powered gates, the proposed NBRFET requires only one control gate and S/D floating gates to achieve the nonvolatile function. Reconfigurable function is realized by programming different types of charges into the S/D floating gates through biasing the gate at a positive or negative high voltage. The effective voltages of the S/D floating gates are determined jointly by the amount of the charge stored in the S/D floating gates and the gate voltage. It should be noted that the gate voltage has a coupling effect on the effective voltage of the floating gates. When the gate electrode is positive biased and the floating gate is already programmed with appropriate amount of positive charges, the effective voltage in the S/D floating gates is higher than the gate voltage. Compared with conventional RFET, NBRFET can achieve higher conduction current under the same gate voltage due to this coupling effect. On the contrary, when the gate electrode is reversely biased, this coupling effect makes the effect voltage of the S/D floating gates lower than the forwardly biased case, then the potential differences between the gate electrode and the S/D floating gates are relatively reduced compared with RFET which has a fixed voltage of program gate, which is conducive to reducing the energy band bending and the corresponding band-to-band tunneling induced leakage current in the reversely biased state. The device scale can be reduced to nanometer level. The device performances such as the transfer and output characteristics and the reconfigurable function are verified by device simulation, which proves that the proposed NBRFET can work better in the nanometer scale.

## Methods

**Fig 1:**
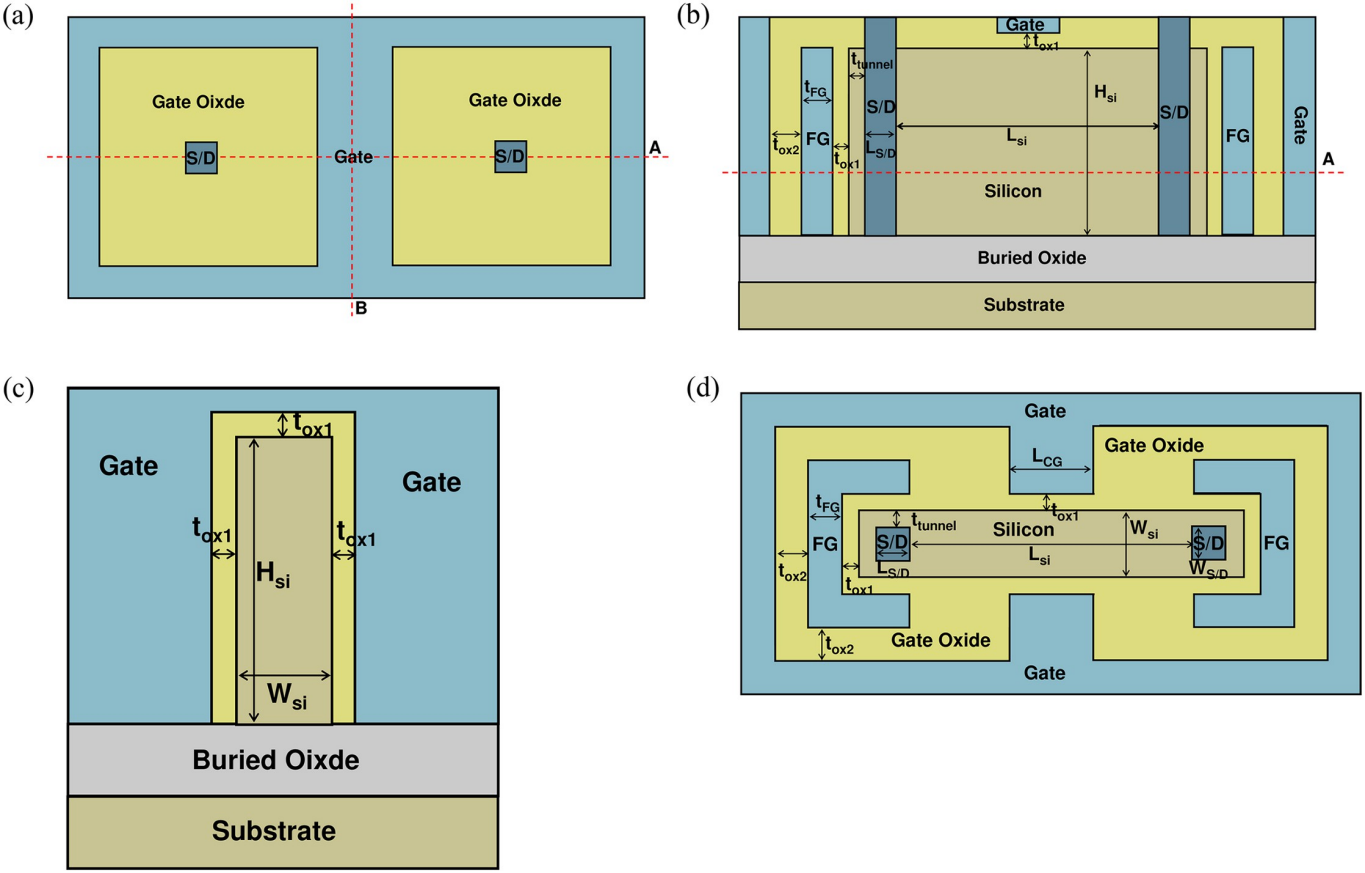
(a) Top view of NBRFET, (b) cross view of NBRFET along cutting line A in Fig 1(a), (c) cross view of NBRFET along cutting line B in Fig 1(a), (d) top view of along cutting line A in Fig 1(b).

Fig 1(a) is a top view of NBRFET, Fig 1(b) is a cross view along the line A in Fig 1(a) and 1(c) is a cross view along line B in Fig 1(a). Fig 1(d) is a top view of NBRFET along line A in Fig 1(b). NiSi is adopted to form Schottky barriers on the interfaces between the source / drain and the silicon. The barrier height between the S/D electrodes and conduction band of silicon is 0.6eV, the barrier height between the S/D electrodes and the valance band of silicon is 0.48eV [17]. L_si_ is the length of silicon between the S/D electrodes, H_si_ is the height of silicon, and W_si_ is the width of silicon. L_CG_ is the length of the control gate in the central region of the device, t_FG_ is the thickness of the floating gate, t_ox1_ is the thickness of the gate oxide between the silicon and control gate / floating gate, t_ox2_ is the thickness of the insulating layer between the control gate and the floating gate on both sides, and t_tunnel_ is the thickness of the tunneling layer between the S/D electrodes and gate oxide.

**Fig 2:**
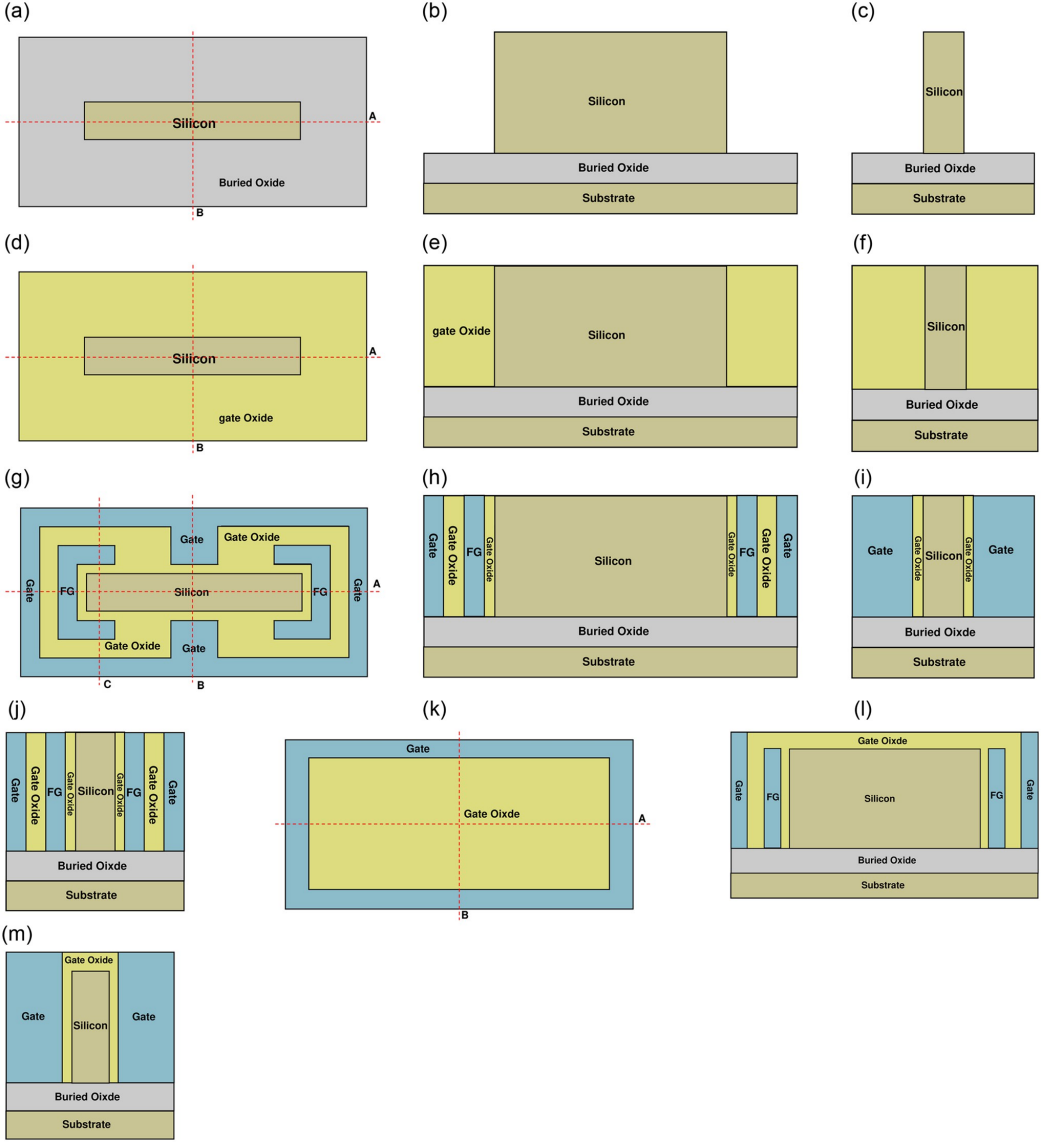
(a) a top view of step1 of fabrication flow, (b) cross view of Fig 2(a) along cutline A, (c) cross view of Fig 2(a) along cutline B, (d) top view of step 2 of fabrication flow, (e) cross view of Fig 2(d) along cutline A, (f) cross view of Fig 2(d) along cutline B, (g) top view of step 3 of fabrication flow, (h) cross view of Fig 2(g) along cutline A, (i) cross view of Fig 2(g) along cutline B, (j) cross view of Fig 2(g) along cutline C, (k) top view of step 4 of fabrication flow, (l) cross view of Fig 2(k) along cutline A, (m) cross view of Fig 2(k) along cutline B.

Device manufacturing is fully compatible with the current CMOS technology. A brief fabrication flow of the proposed NBTFET is shown in Figs 1 and 2. As shown in Fig 2(a), 2(b) and 2(c), prepare a SOI wafer, after removing the surrounding silicon through the photolithography and etching processes, a rectangular silicon area is formed. As shown in Fig 2(d), 2(e) and 2(f), through the deposition process, the insulating dielectric material for the formation of gate oxide layer is deposited. After flattening the surface of the insulating dielectric material through CMP process, the silicon film is exposed again. As shown in Fig 2(g), 2(h), 2(i) and 2(j), remove parts of the insulating dielectric material through photolithography and etching processes, then deposit metal. After flattening the surface of the metal layer to expose the silicon film and gate oxide, both the FG and Gate are initially formed. As shown in Fig 2(k), 2(l) and 2(m), deposit the insulating dielectric material again to further form the gate oxide layer. Then remove insulating dielectric material around through photolithography and etching processes to expose the gate electrode. Then deposit metal again and flatten the surface to expose gate oxide layer, and the gate and gate oxide are further formed. As shown in Fig 1(a), 1(b), 1(c) and 1(d), partially remove the central part of the insulating dielectric material layer, then deposit metal again and flatten the surface to expose the insulating dielectric material layer. The gate electrode is finally formed. Then remove some parts of gate oxide layer and silicon film to expose buried oxide through photolithography and etching processes for reserving space of source and drain electrodes. Then deposit Ni, after annealing, the NiSi interfaces and the Schottky barrier between Ni and silicon are formed on both source and drain sides.

## Analysis and discussions

**Fig 3:**
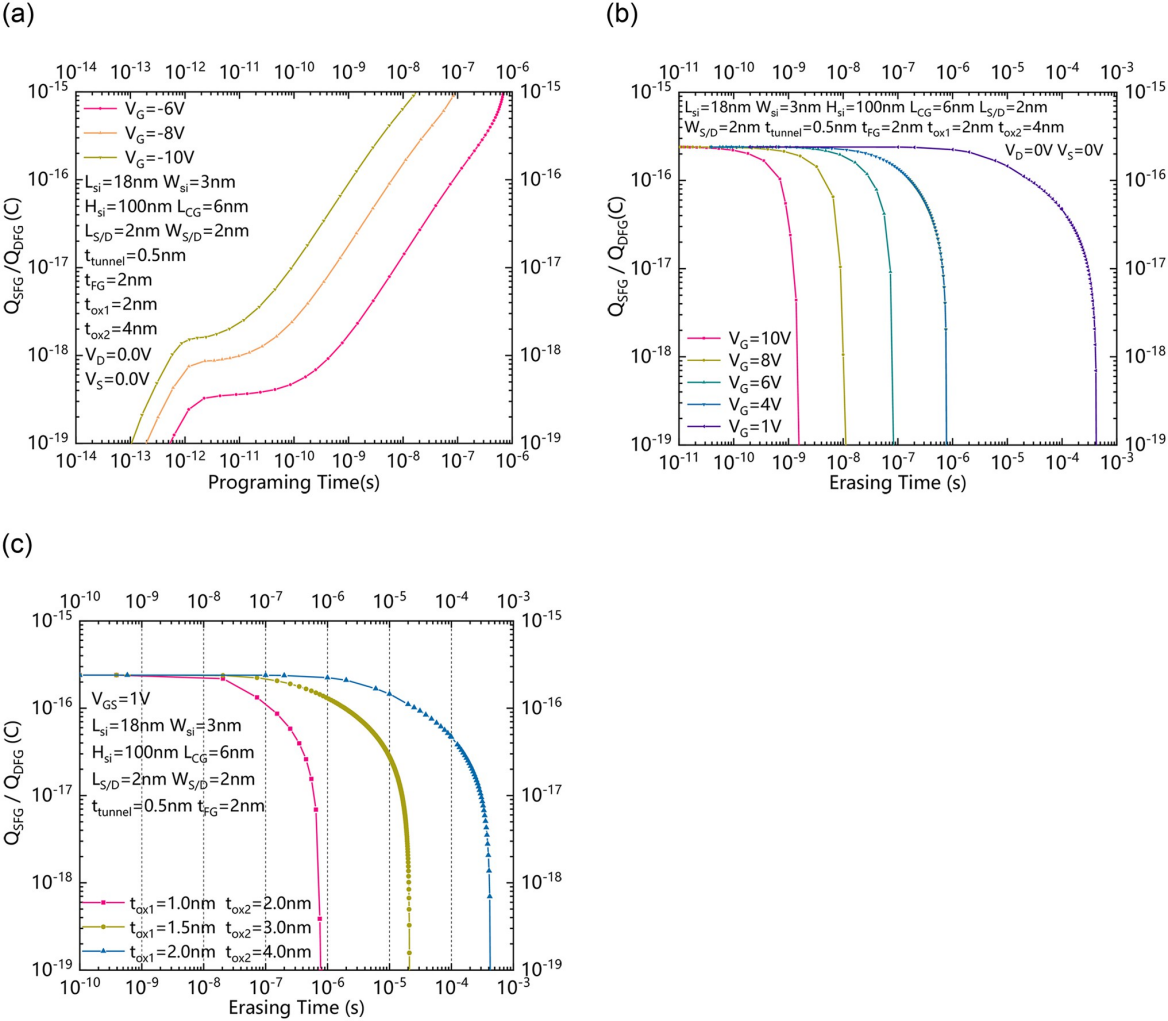
(a) the dependence between the charge stored in the floating gate near the source or the floating gate near the drain (Q_SFG_ or Q_DFG_) and the programming time under different gate voltage V_G_s. (b) the dependence between the charge stored in the floating gate near the source or the floating gate near the drain with an initial Q_SFG_ / Q_DFG_ and the erasing time under different V_G_s. (c) the dependence between the charge stored in the floating gate near the source or the floating gate near the drain with an initial Q_SFG_ / Q_DFG_ and the erasing time under a low V_G_ equals 1V with different t_ox1_ and t_ox2_s.

The performances of the NBRFET and the comparison with conventional BRFET are verified through TCAD simulation [18]. All physics models such as CVT mobility, auger recombination, band gap narrowing, standard BTBT, Fnord tunneling and a compact density gradient quantum confinement model are all turned on. The operation of writing / erasing charge in floating gate is realized by grounding the source electrode and the drain electrode, and simultaneously applying a higher positive or negative voltage to the gate electrode. Fig 3(a) shows the dependence between the charge stored in the floating gate near the source or the floating gate near the drain (Q_SFG_ or Q_DFG_) and the programming time under different gate voltage V_G_s. Q_SFG_ or Q_DFG_ is roughly proportional to the programming time, and the programming speed is roughly proportional to V_G_. Therefore, the programming time can be shortened by applying a higher negative or positive V_G_. Fig 3(b) shows the dependence between the charge stored in the floating gate near the source or the floating gate near the drain with an initial Q_SFG_ / Q_DFG_ and the erasing time under different V_G_s. From the perspective of quantum mechanics, the erasure of electric charges is a probability problem, and increasing V_G_ increases the probability of electric charges being erased. Similar to the programming process, the time required for the first erased charge is also roughly proportional to the V_G_. As a voltage is also required to be applied to the gate electrode during the reading and writing process of the proposed NBRFET, which is often lower than 1V, in order to analyze the impact of the reading voltage on Q_SFG_ / Q_DFG_, we show the dependence between the charge stored in the floating gate near the source or the floating gate near the drain with an initial Q_SFG_ / Q_DFG_ and the erasing time under a low V_G_ equals 1V with different t_ox1_ and t_ox2_s in Fig 3(c). When the thicknesses of t_ox1_ and t_ox2_ are increased, the average time required to erase the first charge in the floating gates will also increase. As Fig 3(c) shows, when t_ox1_ equals to 1 nm, the minimum time required for the erased charge is about 10^−8^ seconds, while when t_ox1_ equals to 2 nm, the minimum time required for the erased charge increases to about 10^−6^ seconds. This means that if the clock frequency of the read signal is greater than 100 MHz for the case that t_ox1_ equals 1 nm, the charge stored in the floating gates will not be easily erased, while if the clock frequency of the read signal is greater than 1 MHz for the case that t_ox1_ equals 2 nm, the charge stored in the floating gates will not be easily erased. For today’s technology, the read frequency is above 1GHz, so even if the thickness of t_ox1_ is as low as 1nm, as long as the read signal clock frequency is high enough, the stored charge in the written floating gate is nonvolatile.

**Fig 4:**
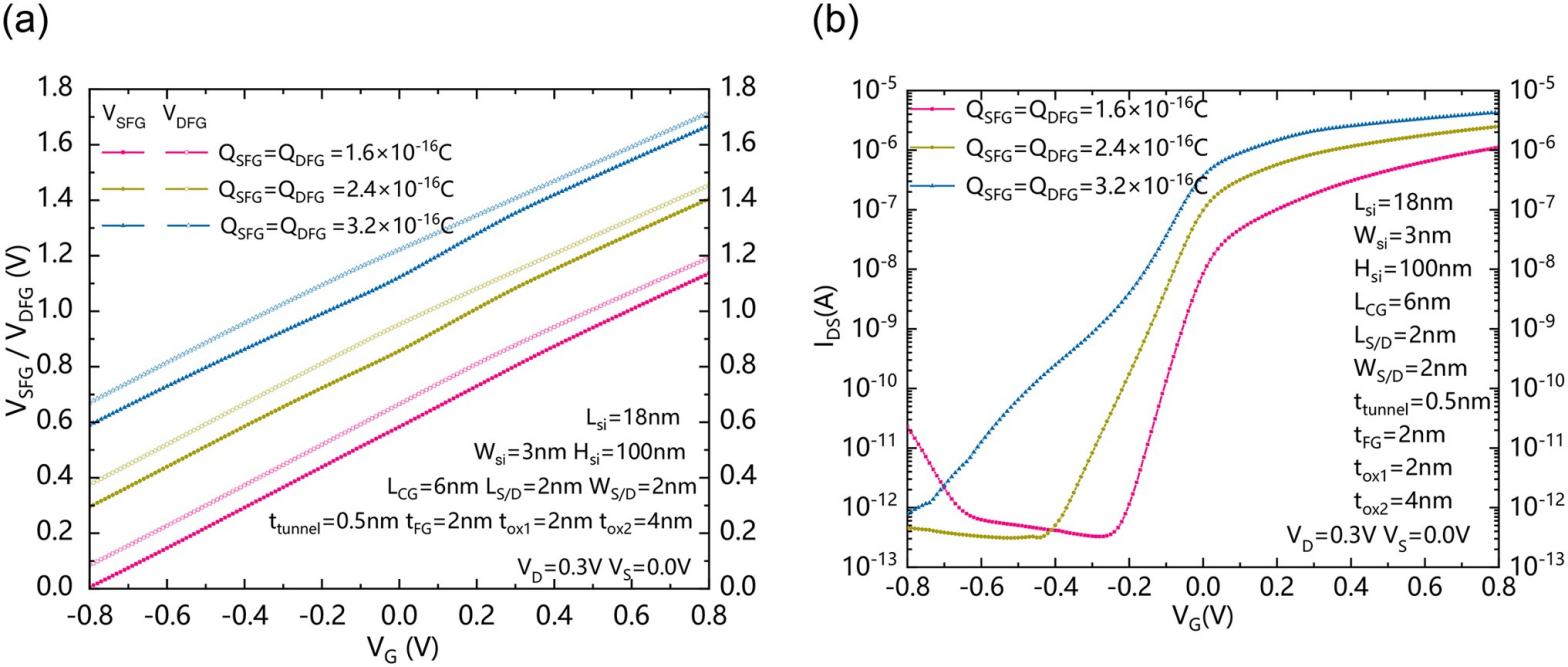
(a) the relationship between the effective voltage of S/D floating gates (V_SFG_ and V_DFG_) and V_G_, and (b) the relationship between the drain to source current I_DS_ and gate voltage V_G_ with different charge stored in the S/D floating gates.

Fig 4(a) shows the relationship between the effective voltage of S/D floating gates (V_SFG_ / V_DFG_) and gate voltage V_G_, and Fig 4(b) shows the relationship between drain to source current I_DS_ and gate voltage V_G_ with different Q_SFG_ / Q_DFG_, respectively. V_SFG_ represents the effective voltage of the source floating gate and V_DFG_ represents the effective voltage of the drain floating gate. In general, the V_SFG_ and V_DFG_ both increase with the increase of V_G_ and decreases with the decreasing of V_G._ Increasing Q_SFG_ / Q_DFG_ is helpful to increase the effective voltages of S/D floating gates in both conditions of forwardly and reversely biased states. For the forward bias state, the V_SFG_ and V_DFG_ both increases due to the increasing of Q_SFG_ and Q_DFG_, leading to more band bending and stronger tunneling effect near the S/D regions in semiconductor. Therefore, the source electrode can provide more carriers from BTBT, so the forward conduction current increases. Due to that there is a coupling effect between V_SFG_ / V_DFG_ and V_G._ Therefore, the V_SFG_ / V_DFG_ can continuously increase with the increasing of V_G._ Therefore, the carriers near the source/drain can be continuously increased. For the reverse bias state, the increase of Q_SFG_ and Q_DFG_ reduces the effective potential difference between the drain electrode and the floating gate electrode, as well as the effective potential difference between the source electrode and the floating gate electrode, so that the energy band bending in the reverse state is greatly reduced. Therefore, it is helpful to eliminate the BTBT effect in the reverse state and reduce the generation of leakage current. As Fig 4(b) shows, there is an optimal Q_SFG_/ Q_DFG,_ it can bring comprehensive improvements in forward current, reverse leakage current, and subthreshold characteristics.

**Fig 5:**
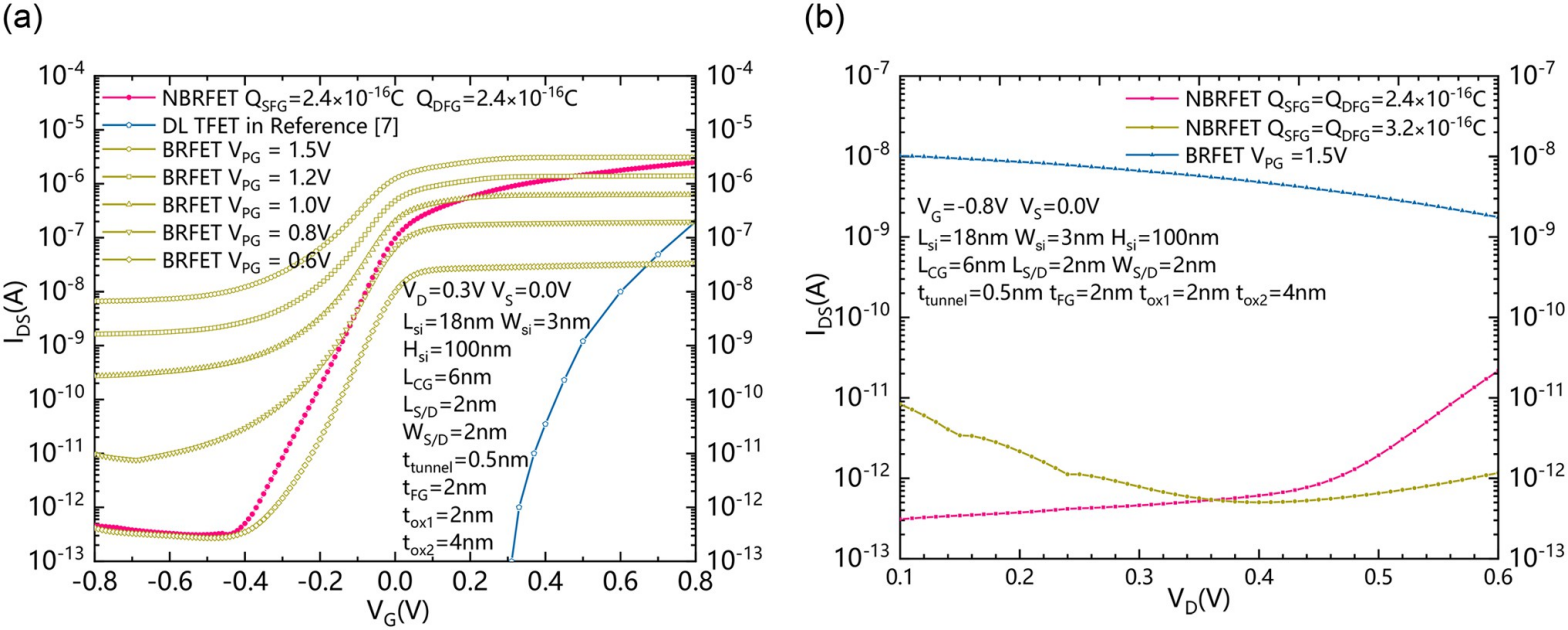
(a) The transfer characteristics comparison between the proposed NBRFET and conventional BRFET with similar geometrical scales. (b) The leakage current comparison between the proposed NBRFET and conventional BRFET with different drain voltage V_D_s.

Fig 5(a) shows the transfer characteristics comparison between the proposed NBRFET, dopingless (DL) TFET in [7] and the conventional BRFET with similar geometrical scales. According to the analysis of Fig 4(a), the effective voltage of V_SFG_/V_DFG_ changes with the amount of charge in SFG and DFG, and they also change with the change of V_G_. There is a coupling effect between V_SFG_/V_DFG_ and V_G_. Therefore, compared to the BRFETs with fixed V_PG_s, the V_SFG_ and V_DFG_ of NBRFET will increase with the increasing of V_G_ and also decrease with the decreasing of V_G_. Therefore, when the gate of NBRFET is reversely biased, V_SFG_ and V_DFG_ have a lower effective voltage. As shown in Fig 5(a), when V_G_ is less than -0.4V, the reverse leakage current of the NBRFET is equivalent to that of BRFET with a V_PG_ of 0.6V. As V_G_ increases, the V_SFG_ and V_DFG_ also increase, the current generated by NBRFET is equivalent to the current generated by BRFETs with larger V_PG_ s. In Fig 5(a), the curves of NBRFET and the ones of BRFETs with different V_PG_ s have an intersection point. Referring to Fig 4(a), the V_SFG_ and V_DFG_ of the NBRFET corresponding to this intersection point are almost equal to the V_PG_ of the BRFET corresponding to the intersection point. It can be clearly seen that the NBRFET with S/D floating gates charged to 2.4×10^-16^C has better forward conduction characteristics and lower reverse leakage current than ordinary BRFET with programming gate with a fixed V_PG_. Fig 5(b) shows the leakage current comparison between the proposed NBRFET and conventional BRFET with different drain voltage V_D_s. It can be seen that the reverse leakage current generated by NBRFET is smaller than that generated by BRFET in a large range of drain voltage.

**Fig 6:**
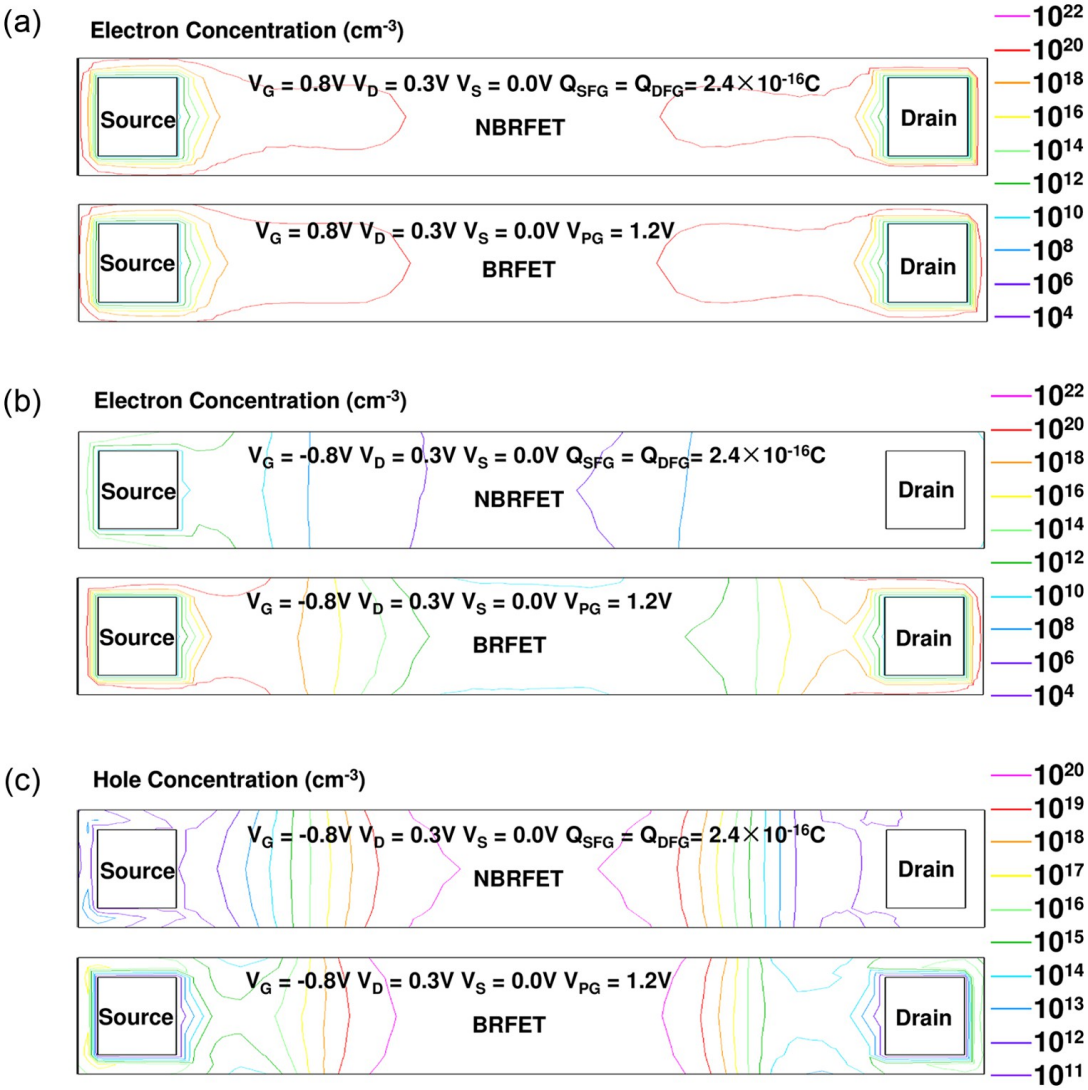
(a) the comparison of electron concentration distribution between NBRFET and BRFET with positively biased gate electrode and positively charged floating gates (b) the comparison of the electron concentration distribution between NBRFET and BRFET with negatively biased gate electrode and positively charged floating gates. (c) the comparison of hole concentration distribution between NBRFET and BRFET with negatively biased gate electrode and positively charged floating gates.

Fig 6(a) shows the comparison of electron concentration distribution between NBRFET and BRFET with positively biased gate electrode and positively charged floating gates. A positive drain voltage V_D_ is also applied. Since the effective voltage in the floating gate is determined by both the type and amount of charges in the floating gate, as well as the gate voltage, the effective voltages of the S/D floating gates increase with the increasing of gate voltage due to coupling effect, thereafter the positively biased gate voltage increase the effective voltage of the S/D floating gates, and the S/D floating gates pulls down the energy band of the silicon regions on both sides of source and drain, and makes the band bending happen through electric field effect near both the source side and the drain side of the silicon region. The band bending induces the BTBT phenomena happen and carriers are generated. The generated holes can be filled by the electrons supplied by the source electrode, the generated electrons flows from the source side to the drain side along the electric field lines generated by the positive V_D_. Thus, the continuous drain to source current is formed. The proposed NBRFET thus works in turn on state. Fig 6(b) shows the comparison of electron concentration distribution between NBRFET and BRFET with negatively biased gate electrode and positively charged floating gates. Fig 6(c) shows the comparison of hole concentration distribution between NBRFET and BRFET with negatively biased gate electrode and positively charged floating gates. The negatively biased gate voltage decreases the effective voltage of the floating gates. The concentration of electron or hole on both sides of source and drain of NBRFET is much smaller than that of BRFET which PG is fixed at a relatively high voltage. Therefore, the source/drain resistance is increased with the decreasing of V_G_. Therefore, as Fig 4(a) shows, the reverse leakage current of NBRFET is as small as that of BRFET with a low PG voltage, while the on-state current of NBRFET is as large as that of BRFET with a high PG voltage.

**Fig 7:**
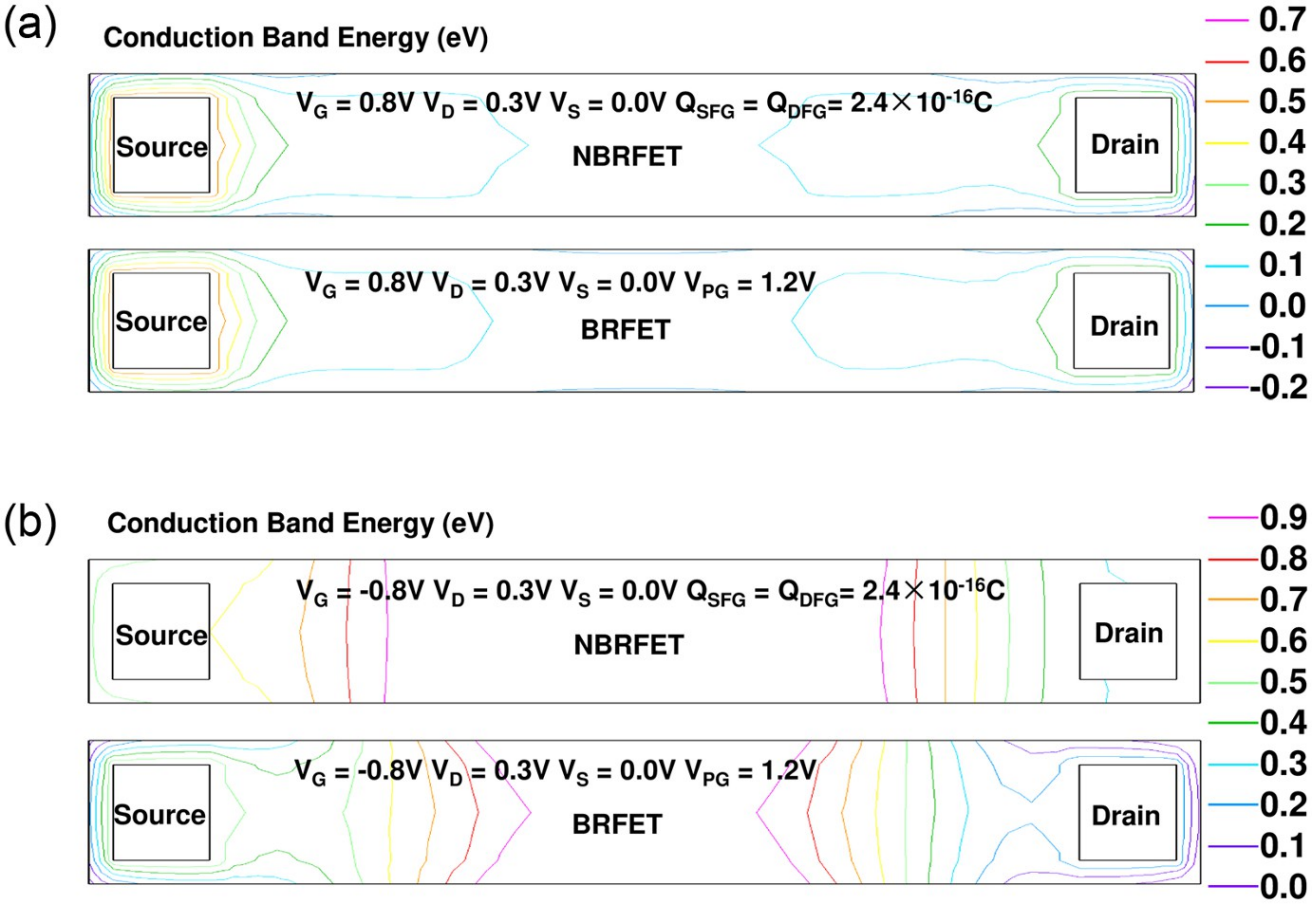
(a) the comparison of conduction band energy distribution between the proposed NBRFET and conventional BRFET in forwardly biased state. (b) The comparison of conduction band energy distribution between the proposed NBRFET and conventional BRFET in reversely biased state.

Fig 7(a) shows the comparison of conduction band energy distribution between the proposed NBRFET and conventional BRFET in forwardly biased state. It can be clearly seen that the energy band contour lines in NBRFET is more intensive. Especially in the tunnel layer, the band bending amplitude reaches 0.45eV. For the BRFET, because its fixed programmed gate voltage does not change with the change of the gate voltage, the band bending amplitude is maintained at 0.35eV. This is the fundamental reason why the forward conduction current of NBRFET can be continuously increased by increasing V_G_, while the forward conduction current of BRFET will saturate for a large V_G_. Fig 7(b) shows the comparison of conduction band energy distribution between the proposed NBRFET and conventional BRFET in reversely biased state. It is also clear that due to the effective voltage of S/D floating gate decreases with the decreasing of gate voltage, the energy band contour distribution near the source/drain region in NBRFET becomes very sparse compared to the conventional BRFET, therefore, NBRFET can achieve much lower reverse leakage current under the same reversely biased gate voltage.

**Fig 8:**
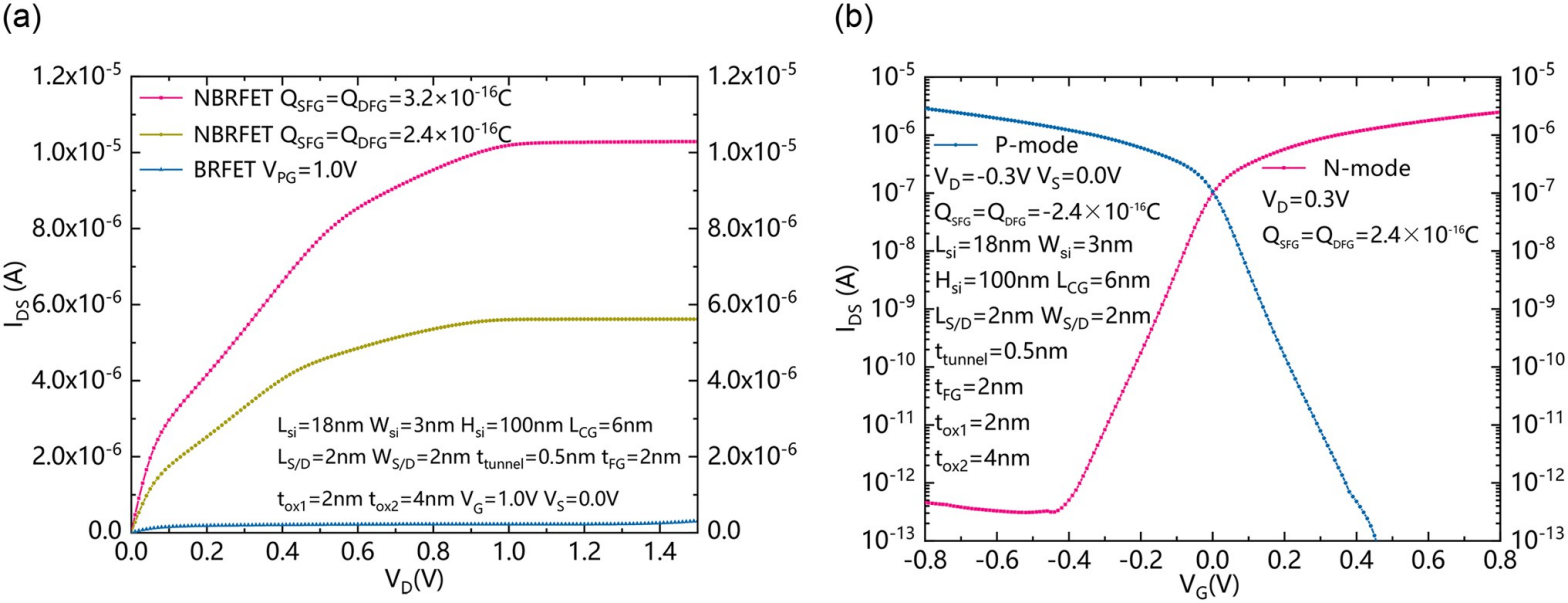
(a) The output characteristics between I_DS_ and V_D_. (b) The reconfigurable characteristics of the proposed NBRFET.

Fig 8(a) and 8(b) show the output characteristics between I_DS_ and V_D_, and the reconfigurable characteristics of the proposed NBRFET, respectively. As shown in Fig 8(a), the output characteristics I_DS_ is increasing with the increase of Q_SFG_ and Q_DFG_. Compared with the conventional BRFET, the proposed NBRFET can obtain better output characteristics, when the S/D floating gates are stored with appropriate amount of charge. Fig 8(b) shows that the charge type stored in the floating gate can determine the conduction mode of the proposed NBRFET. When the S/D floating gates are stored with positive charge and the gate is also forwardly biased, electrons in the conduction band generated by the BTBT effect can flow to the drain electrode and the device operates in the turn-on state of n-mode. Similarly, when the S/D floating gates are stored with negative charge and the gate is reversely biased, the valence band holes generated by the BTBT effect can be allowed to pass through, and the device operates in p-mode.

**Fig 9:**
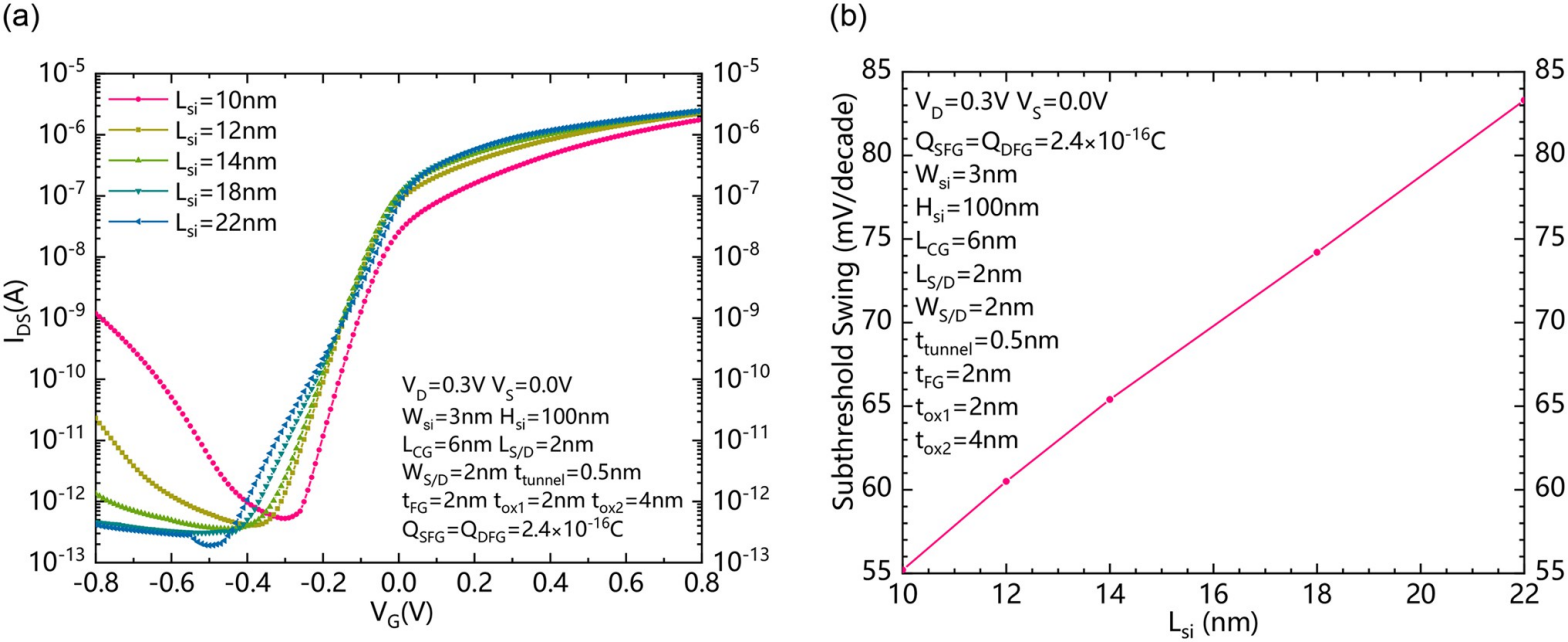
(a) the I_DS_-V_G_ dependence of NBRFET with different L_si_s. (b) The dependence between subthreshold swing and L_si_ of NBRFET.

Fig 9(a) shows the I_DS_-V_G_ dependence of NBRFET with different L_si_s. Fig 9(b) shows the dependence between subthreshold swing and L_si_ of NBRFET. As the distance between FG and gate decreases with the decreasing of Lsi, when the gate is reversely biased, the energy band bending between FG and Gate is also enhanced, therefore the reverse leakage current increases with the decreasing of Lsi. However, as the Lsi decreases, the gate controllability to the whole channel from source to drain is also increased. Therefore, as Fig 9(b) shows, the SS decreases with the decreasing of L_si_s. There is a Compromise relationship between the reverse leakage and SS. The recommend optimal Lsi is about 14nm.

**Fig 10:**
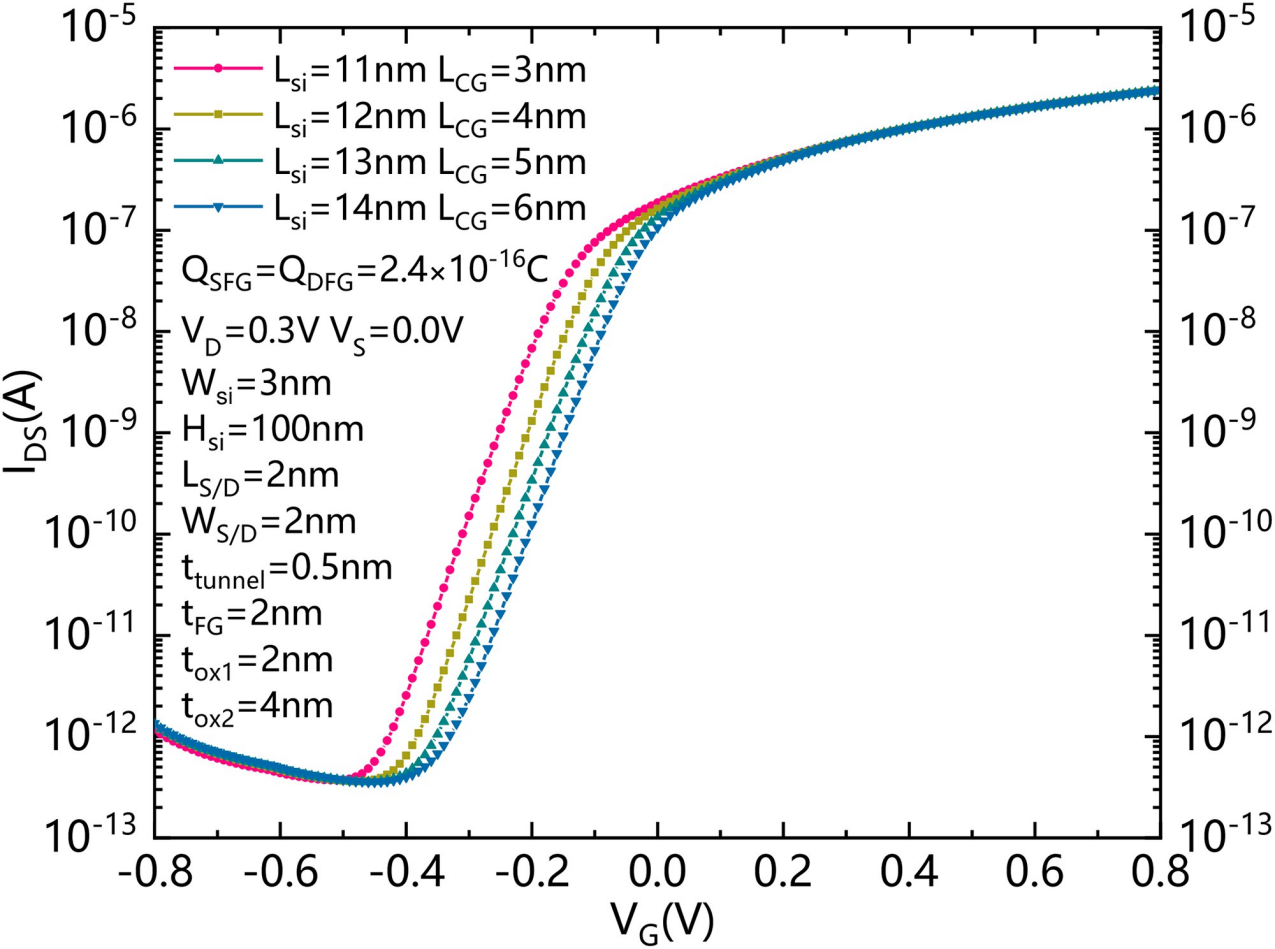
The I_DS_-V_G_ dependence of NBRFET with different L_CG_s and L_si_s.

Fig 10 shows the I_DS_-V_G_ dependence of NBRFET with different L_CG_s and L_si_s. As L_CG_ decreases, the controllability of Gate to the channel potential is weakened. Lower reverse VG is needed to cut off the channel of electrons. Therefore, changes in the L_CG_ have an impact on the threshold voltage. However, this change has little effect on the forward conduction current and the reverse leakage current.

## Conclusions

In this paper, we propose a nanoscale nonvolatile bidirectional reconfigurable field effect transistor (NBRFET) based on source /drain (S/D) self programmable floating gates. Different from the conventional RFET which requires two independently powered gates, the proposed NBRFET requires only one control gate and introduces S/D floating gates to realize the nonvolatile function. Reconfigurable function is realized by programming different types of charges into the S/D floating gates through biasing the gate at a positive or negative high voltage. The effective voltages of the S/D floating gates are determined jointly by the amount of the charge stored in the S/D floating gates and the gate voltage. In addition, the charge stored in the floating gate has an effect of reducing the energy band bending near the source/drain regions when the gate is reversely biased, thereafter, the BTBT leakage current can be largely decreased. The device scale can be reduced to nanometer level. The device performances such as the transfer and output characteristics are verified by device simulation, which proves that the proposed NBRFET has very good performance in the nanometer scale.

## Supporting information

S1 File (XLS)

S2 File (XLS)

S3 File (XLS)

S4 File (XLS)

S5 File (XLS)

S6 File (XLS)

S7 File (XLS)

S8 File (STR)

S9 File (STR)

S10 File (STR)

S11 File (STR)

S12 File (XLS)

S13 File (XLS)

S14 File (XLS)

S15 File (XLS)

S16 File (XLS)
